# A Review on Orthodontic Brackets and Their Application in Clinical Orthodontics

**DOI:** 10.7759/cureus.46615

**Published:** 2023-10-07

**Authors:** Vedanti V Mundhada, Vikrant V Jadhav, Amit Reche

**Affiliations:** 1 Dentistry, Sharad Pawar Dental College and Hospital, Datta Meghe Institute of Higher Education and Research, Wardha, IND; 2 Orthodontics and Dentofacial Orthopaedics, Sharad Pawar Dental College and Hospital, Datta Meghe Institute of Higher Education and Research, Wardha, IND; 3 Public Health Dentistry, Sharad Pawar Dental College and Hospital, Datta Meghe Institute of Higher Education and Research, Wardha, IND

**Keywords:** dentists, metal braces, orthodontists, mbt brackets, dental bracket

## Abstract

Braces are undoubtedly a blessing for those whose teeth are misaligned in various ways, yet many people may not enjoy the traditional metal braces. However, some people are totally fine with having their teeth fused with the set of metal brackets and various metal components flowing through. A few people may simply not feel comfortable in that way. Orthodontists have developed a huge array of bracket styles by concentrating on the wide range of preferences and financial constraints that their patients present. These various dental bracket types are all advantageous from one point of view or another. Orthodontic supplies and materials are being produced using technology that is advancing exponentially. Every day, more technologies, processes, and designs emerge. These tools assist the orthodontist in providing patients with the best functional and cosmetic outcomes. Since Edward Hartley Angle's time through MBT brackets and then lingual brackets, orthodontic brackets have advanced significantly. Orthodontists' lives have been greatly facilitated by these brackets. Numerous new materials and designs will be developed as technology progresses. The purpose of this article was to give a general overview of the different orthodontic brackets and demonstrate how the logic underlying them helps orthodontists in their day-to-day jobs.

## Introduction and background

The human race has benefited greatly from orthodontics. Since the first half of the 18th century, orthodontics has been a field of study. To understand those procedures, today's orthodontists looked to generations of their forebears. The rate of technological advancement in orthodontic therapy is astounding. Orthodontists may now provide better experiences to their patients with less difficulty owing to recent discoveries and inventions. We must make sure that we keep up with developments in the industry if we want to continue offering our patients prompt, effective care. From the era of Edward Hartley Angle, the acknowledged father of modern orthodontics, which was from 1899, the orthodontic brackets have advanced, first giving way to the McLaughlin, Bennett, Trevisi brackets system and then lingual brackets. Orthodontists' lives have been significantly simplified by these brackets. Patients seeking orthodontic treatment, including an increasing number of adults, need superior aesthetics in addition to an enhanced smile. The presence of fixed orthodontic equipment has historically caused a lot of patients to express specific anxiety. It has remained a challenge to create appliances that offer both the patient's desired aesthetics and the orthodontist's required technical performance. The various approaches that are been tried to meet this criterion include modifying stainless steel brackets' complexion or lowering their size, shifting the appliance toward the lingual sides of teeth, and modifying components used for creating the bracket. Small stainless steel brackets are becoming more and more common, but although typically match the orthodontist's standards for technical efficiency, they do not provide many cosmetic advantages over equipment of the same size [1,2].

After stainless steel brackets, the lingual bracket system was introduced. Although lingual orthodontic bracket seems aesthetically convincing, it is contended that they result in a reduction in the performance of the appliance and much more practical challenges and time demands for orthodontists [3]. In late 1986, the initial ceramic-based bracket became commercially available, which was again more aesthetically pleasing. Patients have rightfully embraced ceramic brackets since they represent the most efficient attempt to date in creating an orthodontic appliance that satisfies both the patient's aesthetic needs and the orthodontist's technical performance requirements [1]. Ceramic brackets have undeniably better aesthetics than stainless steel ones, but this is still the sole advantage they have over stainless steel because of the mechanical issues that arise when using them in clinical settings [4]. Numerous new bracket generations are emerging on the market. One must be up to speed on all the latest advancements such as 3D imaging, digital impressions, and the advances in bracket design so as to give patients the best practical and cosmetic results.

## Review

Method

**Figure 1:**
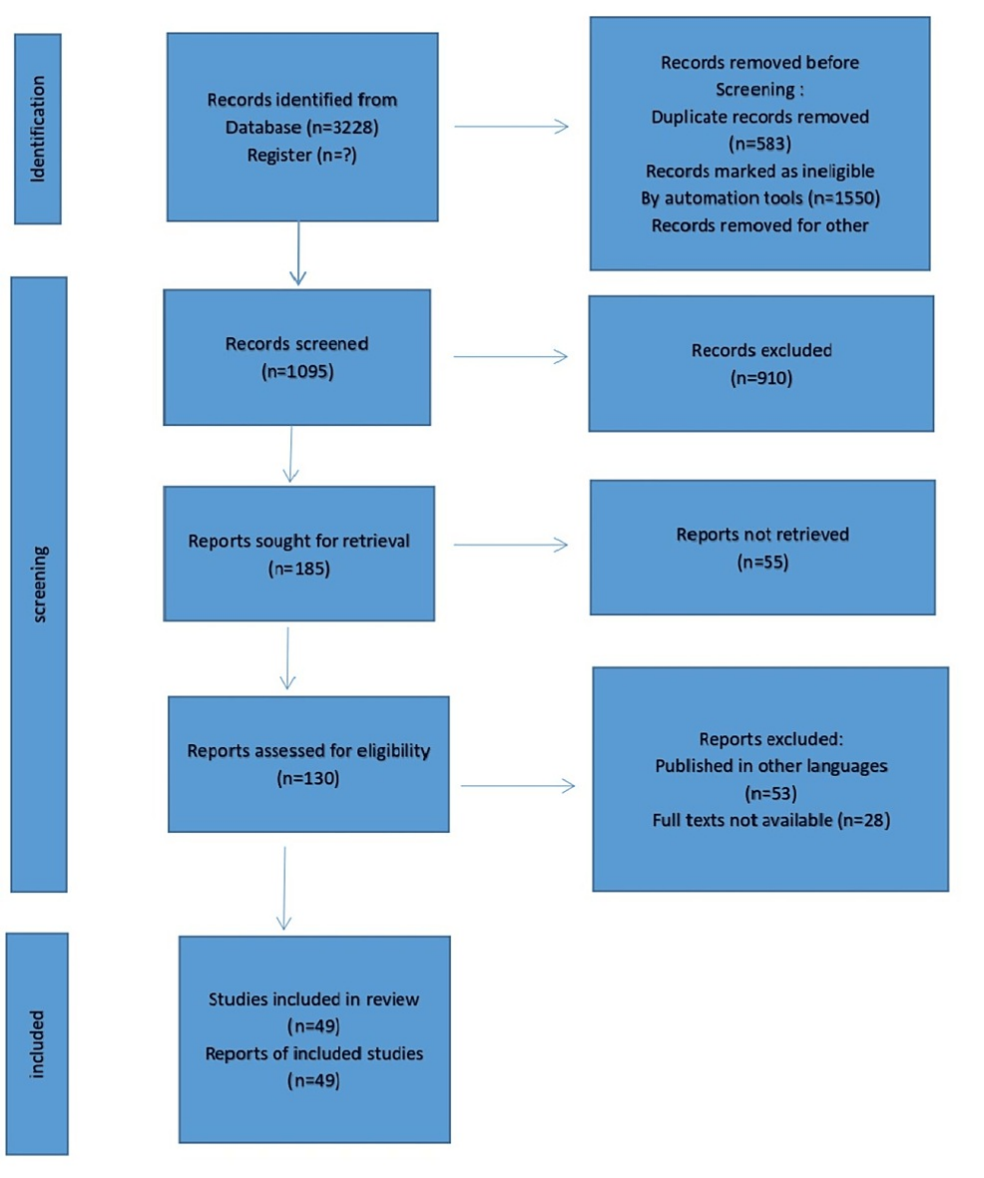
The selection process of the articles used in this study

We undertook a systematic search through PubMed and Google Scholar in October 2022 using keywords such as “dentist”, “metal braces", "orthodontists", "brackets”, OR “Orthodontics". We additionally searched for key references from bibliographies of relevant studies. The search was updated in January 2023. One reviewer independently monitored the retrieved studies against the inclusion criteria, in the beginning, based on the title and abstract and then on full texts. Another reviewer also reviewed approximately 1.52% of these studies to validate the inclusion of studies. For inclusion, both published and unpublished studies in the English language were considered. We excluded studies that were published in other languages because of resource limitations or if the full-text articles were unavailable to reviewers. We excluded case reports corresponding to the letter to the editor. Figure 1 presents the selection process of articles used in this study.

Stainless steel (SS) brackets

**Figure 2:**
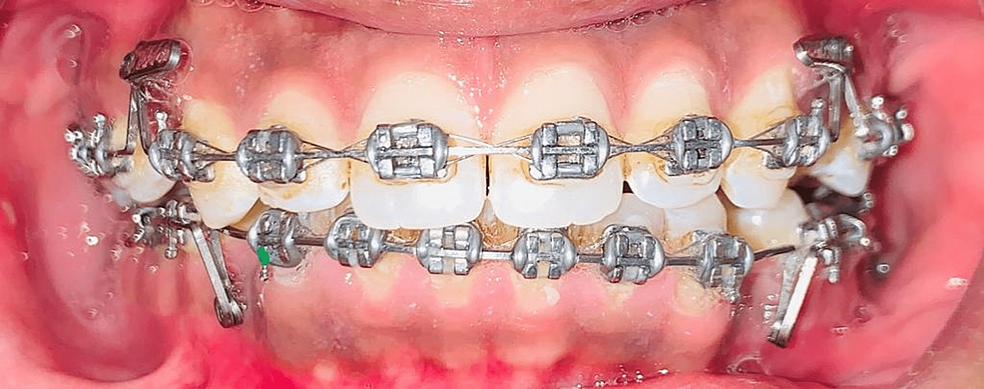
Stainless steel brackets Image Credits: The image is taken by one of the authors

These are the greatest brackets available in terms of affordability and durability, in addition to being the most widely used metal brackets. Some of its other advantages include high stiffness, high yield strength, high resilience, biocompatibility, and corrosion resistance. From the beginning, orthodontists have employed these SS brackets. Most of the metal brackets are made up of stainless steel (SS) [5]. Figure 2 shows SS brackets. It has certain drawbacks as it demands soldering, aesthetically non-pleasing, high modulus of elasticity, more frequent number of activations, and low springback than NiTi alloy, and heating it to temperatures between 400 and 900 degrees causes the release of Ni and Cr, thereby decreasing corrosion resistance. Attempts to recycle brackets have subsequently been made. Smith [5] demonstrated that the bond strength and corrosion resistance were significantly reduced. Super SS is categorically defined as SS with a pitting resistance equivalent (PRE) value greater than 40. The super SS (SR-50A) employed in this work has localized corrosion resistance that is as excellent as titanium alloys (6.77%) because of the synergistic impact of heavy concentrations of nitrogen (0.331%) and molybdenum (0.904%) and is also believed to have high mechanical characteristics because of a "solution-strengthening effect" [6].

Ceramic brackets

**Figure 3:**
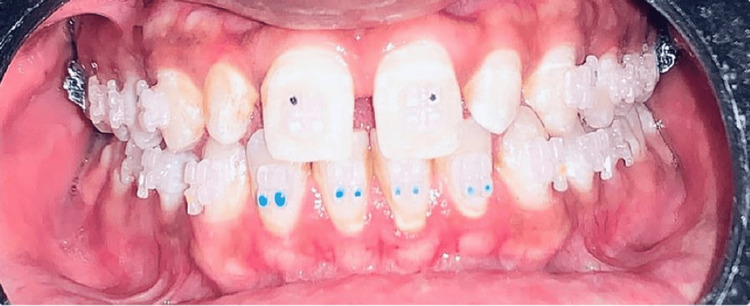
Ceramic brackets Image Credits: The image is taken by one of the authors

Ceramic brackets were first launched in the 1970s and provide a number of benefits over conventional cosmetic accessories. Ceramics are made of components that are initially created and then heated until they become firm. They can be difficult to spot at first as they gradually turn a little yellow. These could be your preferred braces if you cannot stand the thought of having metal on your teeth. Ceramic braces are available in various morphologies, such as solid, Lewis/Lang, real Siamese, and semi-Siamese designs, as well as in a range of appliance systems, such as Begg bracket and ligation brackets with variable force [1]. Ceramic brackets, also referred to as attractive brackets, are favored for their translucent appearance. Figure 3 shows ceramic brackets. Ceramic braces are of benefit in various ways as they offer more strength, increased durability, improved stability in case of color, and efficient aesthetic quality as compared to SS brackets. Its shortcomings include a lack of ductility, being expensive, fragile, staining easily, bulky, and a complicated and expensive manufacturing process. The ceramic material utilized in orthodontic brackets is alumina, and depending on how they are made, they can be either polycrystalline or monocrystalline. Extrusions of sapphire single crystals are used to fabricate monocrystalline brackets. Contrarily, submicron-sized polycrystalline sapphire (alumina) particles floating in a resin are used for creating polycrystalline brackets of alumina, which are then machined as needed to create the final product. According to the hardness statistic compared to stainless steel, both monocrystalline and polycrystalline alumina offer a significant benefit. In terms of tensile strength, monocrystalline alumina is significantly stronger than polycrystalline alumina, giving it a major benefit over steel. Alumina, whether monocrystalline or polycrystalline, has poor fracture toughness and thus has resistance to crack propagation when compared to SS [1].

The optical clarity of polycrystalline brackets as opposed to single crystal brackets is the primary distinction. Brackets made of a single crystal are translucent because they are notably clearer than brackets made of many crystals. Fortunately, single-crystal and polycrystalline brackets are resistant to stains and fading [7,8]. Alumina ceramic brackets have been replaced by polycrystalline zirconia brackets (ZrO), which supposedly have the highest toughness of any ceramic [9]. Compared to monocrystalline ceramic brackets, they are less costly, but because of their high opaqueness and potential for intrinsic coloration, they are less aesthetically pleasing [10]. There have been reports of clinically satisfying bond strength, decreased plaque adherence, favorable sliding characteristics, and bond failure locations at the bracket/adhesive interface for both SS and nickel-titanium archwires. Zirconia brackets did not beat polycrystalline alumina brackets in terms of frictional characteristics, according to Keith et al. [4]. Since alumina ceramic brackets routinely outperformed them in clinical settings, they are no longer employed and will only be taken into account moving forward [4]. Utilizing a zirconia fireworking technique that may impart lubricating qualities to the internal surface of the wire slots and the outside surface of the brackets, Maki et al. [11] constructed a unique orthodontic bracket with three slots. Additionally, they suggested a novel approach to orthodontic therapy with 0.012-0.014-inch NiTi archwires. In the early stages of leveling, the proposed innovative orthodontic treatment technique demonstrated a faster tooth movement rate, and the mean period of treatment until leveling was greatly shortened [11].

Plastic brackets

Plastic brackets are another variety of brackets used in orthodontics and offer comfort and cosmetic appeal simultaneously. To match the patient's teeth, the plastic fibers are tinted. Aside from that, the discomfort will also be greatly reduced by their soft edges. Nevertheless, they could not be as strong as other kinds of orthodontic brackets. In the first decade of the 1980s, plastic brackets were promoted. They were only briefly used by dentists as a beautiful substitute for metal braces. They were initially made of acrylic and later polycarbonate. In addition to other problems that were quickly identified, the materials' absence of stiffness and strength led to bonding problems, tie wing fracture, and irreversible warping. Odors and discoloration were among these problems [1]. In a simulated intraoral setting, Harzer et al. [12] revealed that, compared to metal brackets, polycarbonate brackets had larger torque losses and lower torquing moments. High-grade medical polyurethane and polycarbonate brackets reinforced with ceramic or fiberglass fillers and/or metal holes have lately been created and are gaining popularity as a solution to the stiffness and strength difficulties of conventional polycarbonate brackets.

Although torque issues still exist, polycarbonate brackets with metal-reinforced holes exhibit less creep than conventional polycarbonate brackets. Metal-lined and ceramic-reinforced polycarbonate brackets both demonstrated a 15% reduction in torque over the course of a day. These brackets function better than polycarbonate brackets, although they could be able to compete with ceramic brackets in the future [12] with more study. Sadat-Khonsari et al. [13], while evaluating the torque deformation characteristics of seven commercially available plastic brackets to SS brackets, found that pure polyurethane, pure polycarbonate, and fiberglass-reinforced polycarbonate brackets had the least distortion. The brackets made of ceramic-reinforced polycarbonate exhibited the highest degree of deformation when put under torque loads. Even while pure polyurethane brackets did not considerably vary from pure polycarbonate at ideal torque, the adding of ceramic and fiberglass to plastic brackets did not significantly boost the torque stability of the polycarbonate brackets. When compared to SS brackets, plastic brackets are only appropriate for use in clinical settings when they have a metal groove so as to prevent breakage as it increases the strength of the bracket [13].

Self-ligating brackets

**Figure 4:**
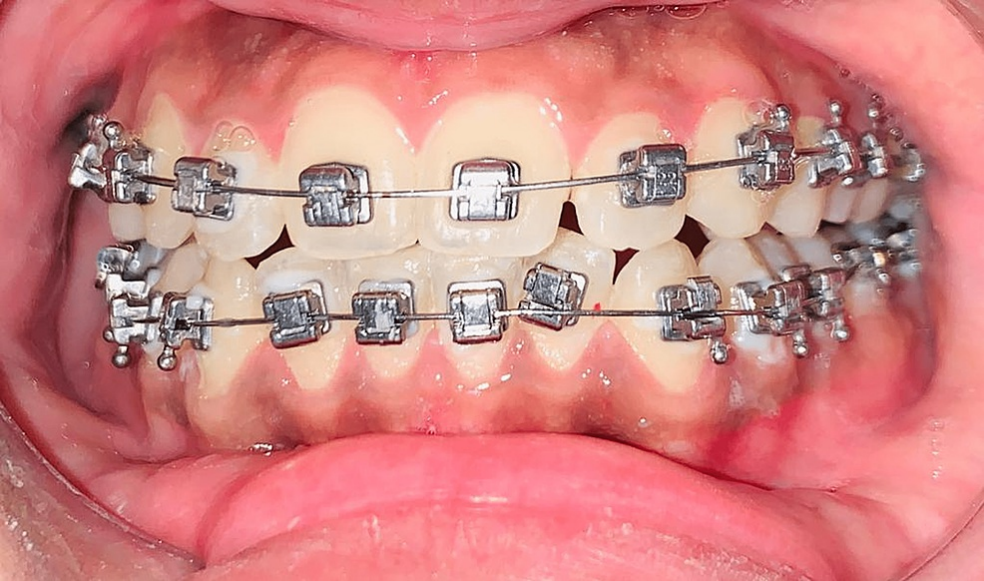
Self-ligating brackets Image Credits: The image is taken by one of the authors

In the first decade of the 1930s, Stolzenberg created the Russell attachment, the pioneering self-ligating bracket. In comparison to traditional edgewise brackets, these self-ligating brackets are said to have many benefits [14-16]. There are various materials available for these brackets. Patients have the option of choosing a metallic as well as ceramic one, but this is not their specialty. Regular adhesive or elastic bands are not required for these brackets. Their unique shape provides the necessary mobility and tension simultaneously. They can be divided into active and passive types based on how they close. Active self-ligating brackets exert an active force through a spring clip onto the arch wire in order to maintain it in the slot, whereas passive self-ligating brackets present an additional slide that once closed does not affect the slot lumen, nor exert an active force on the wire. The passive self-ligating brackets generate significantly larger torquing moments, giving better performance. They are said to have less friction than traditional brackets, which is frequently highlighted as one of their main advantages, making them increasingly popular [15,17-20]. This happens because the typical ligatures made of steel or elastomer are not required, and it has been asserted that the passive design produces significantly lesser friction compared to the active one [20,21]. Figure 4 shows self-ligating brackets. They have minimal friction and consequently require less force to move teeth [22]. They are thought of as having the ability to generate tooth movement that is more physiologically harmonious by preventing the musculature from being overworked and the periodontal vascular supply from being cut off [15]. It is believed that, as a result of physiological movement, it is possible to generate more alveolar bone, expand the jaw further, reduce anterior tooth proclination, and reduce the need for extractions. Additionally, full and secure wire ligation [23], enhanced sliding mechanics and potential anchorage preservation [14,24], extended treatment intervals with very couple visits [14,25,26], chair time is saved, reduced chair-side assistance, superior overall ergonomics [22,26-28], enhanced control of infection [26], reduced distress to patients [14,16], and optimized oral hygiene [27-29] are all touted benefits. However, there are a few drawbacks. For example, due to their sophisticated mechanical design, there may be greater occlusal discrepancies and lip pain; they are more expensive, have a larger profile, and are difficult to complete as a result of archwires' partial expression; and there is a chance that a clip or slide will break, among other factors.

Harmony Lingual Self‑Ligating Bracket System (2011)

Its framework produces fully changed bonding pads and structurally formed archwires that effectively and accurately move teeth [30].

Sensation Active Ceramic Self-Ligating Brackets (2012)

It is made of a sturdy, transparent ceramic material and features a steel clip with rhodium plating. Faster archwire changes result from a unique guide rail that balances the bracket clip's opening and closing pressures [31].

Bioquick Self‑Ligating Brackets (2014)

So as to improve patient comfort, Forestadent's BioQuick self-ligating bracket now has a reduced profile and rounded edges. The improved clip now has a 20% thicker wall, increasing its strength and ability to withstand damage while also providing superior control over angulation, rotation, and torque [32].

Carriere SLX Self‑Ligating Bracket System (2014)

An improved version of the Damon solution is provided by Henry Schein Orthodontics' new Carriere SLX self-ligating bracket system, which has improvements in bracket placement, torque control, and precision finishing. The bracket has occlusal opening doors and an impressively low profile. Visual cues, including six horizontal and five vertical references, are meant to aid in ensuring proper bracket placement [33].

Empower 2 (2016)

Empower 2 is a modernized version of the Empower self-ligating bracket system. Micro-etched bonding pads, designed to increase binding strength by 15%-30% over varied bases, are among the new features. Another is a larger clip, which increases wire retention strength while preventing clip deformity [34-37].

In‑Ovation X (2017)

Dentsply Sirona's most recent addition to its self-ligating is Ovation X. It maintains the same basic structure and treating criteria but has been improved with a more streamlined design, a smaller profile, and a reduced occlusal impression. A newer enclosed clip system and closed gingival bracket basis will lessen calculus growth, which might interfere with clip performance [38,39].

Lingual brackets

**Figure 5:**
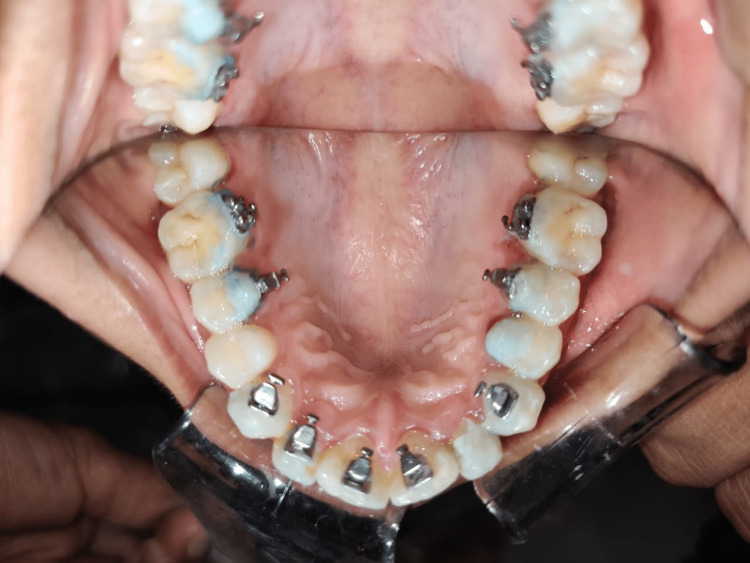
Lingual brackets Image Credits: The image is taken by one of the authors

Lingual brackets are the same as various metallic orthodontic bracket kinds. The primary difference is that they are attached to the back of your teeth, not the front. Figure 5 shows lingual brackets. For those who prioritize aesthetics, this is particularly appealing. In addition, they protect your teeth from over-calcification. The lingual approach with a mushroom-shaped archwire had first been popularized by the Fujita. He started researching on lingual method in 1968, continued with studies in 1971, and eventually released the ideas behind the Fujita Bracket in 1978 [40,41]. The preferred outcome of adults and rising interest in lingual orthodontics, as well as the aesthetic and completely hidden nature of the device, are its benefits [41,42]. Speech disruption, occlusal interferences that regularly led to bond breakdowns and occasionally inhibited tooth development, an indirect vision that made it challenging to set brackets accurately, tongue laceration from sharp edges, gingival irritation from plaque build-up, and longer chair-side time due to the difficulty of inserting and ligating the archwire are some disadvantages.

Butterfly system

It is constructed on a brand-new, vertically slotted, low-profile bracket. The vertical slot makes the product more adaptable by allowing the attachment of various auxiliary devices. They can be inserted anytime over the course of therapy when hook or T pins for elastics are required. As a result, there is no longer a requirement for brackets to be produced with hooks [43]. The creation of the Butterfly system started off by utilizing Andrew's brilliant ideas [44].The lowered profile or thickness of the bracket, its tiny Siamese twin design, and its curved tie wings all contribute to the patient's convenience and attractiveness. An appliance that combines these attributes with the removal of hooks is much more pleasant, attractive, and tidy [44,45]. Although further enhancements could be beneficial in order to achieve ideal tooth positions with the help of straight wires because there can be inaccurate bracket placement, variation in tooth structure, variations in antero-posterior jaw relationships affecting incisor position, and a lack of overcorrection built into treatment mechanics or mechanical deficiencies of orthodontic appliance. Table 1 shows six distinct elements intended to enhance pre-adjusted appliance designs now in use.

**Table 1:** Six distinct elements intended to enhance pre-adjusted appliance designs now in use Source: Ref [44]

Developing posterior torque
Second premolar angulation that can be reversed
Mandibular anterior torque prevention
Progressive forward angulation of the mandible
Pre-welded convertible molar tubes with a six-degree angulation
Increased adaptability for extraction as well as non-extraction therapies

Gold brackets

In the past, numerous people have already been drawn to braces that had the least visible appearance. Usually, dental experts do this by placing the braces behind patients’ teeth or using materials that are transparent or tooth-colored. Certain individuals like to draw attention to their braces and make them a distinctive, eye-catching feature of their appearance. Gold braces could be a good choice for those who describe this. Since 2002, gold lingual brackets have been utilized in orthodontic clinical practice; however, it is unknown if their technical or chemical qualities have changed as a result of intraoral use [46]. One little way that gold braces vary from traditional silver braces is that the stainless-steel parts are gold-plated. This gives the illusion that the wire holding the brackets to the teeth and the brackets themselves are both made of gold. Due to the fact that gold is thought to be more fashionable, attractive, and, for some, a symbol of high socioeconomic standing, some individuals prefer the appearance of gold braces above regular braces. Beyond cosmetic considerations, gold braces have additional advantages since they are anti-inflammatory and less prone to trigger allergic reactions. IncognitoTM is a well-known completely personalized lingual equipment technology that was introduced in 2004 [47]. Because they are made of a high-gold alloy without nickel, these brackets can be used to treat those who have nickel hypersensitivity [48,49]. The first step should be to consult your dentist to discuss your alternatives and figure out exactly how much they will cost you. Gold braces are more expensive than conventional braces and are not provided by all orthodontists.

Silver platinum-coated brackets

Contrary to other types of medical equipment, chewing food can corrode or abrade orthodontic gadgets when used in oral settings. Palladium and other members of the platinum family elements are frequently utilized in alloys because they are chemically resistant. Palladium has reportedly been added to the Ag plating process to increase hardness and resistance to wear [50]. On surfaces comprising load-bearing orthodontic appliances, Ag-Pt coatings can offer effective antibacterial activity and tolerance to biofilm development. For orthodontic patients, particularly those with poor periodontal health or high caries risk, brackets or archwires with this Ag-Pt covering can be produced [51].

Titanium brackets

Because of their physical strength and ease of shaping into diverse forms, metallic materials make up the majority of the bracket being used in orthodontic mechanotherapy. Concern has recently grown over patients' adverse responses to metallic intra-oral prostheses. Numerous publications, in particular, have discussed clinical examples of nickel as well as chromium hypersensitivity [9,52]. After injection molding, pure titanium powder was sintered to create titanium brackets. Enamel bonding and hardness tests were performed on the manufactured brackets in order to compare them to the SS bracket used as a control. The efficiency of arch-guided tooth movement would be enhanced by the material's superior biocompatibility and lower friction [53]. However, compared to steel brackets, titanium bracket exhibits more plaque build-up and more discoloration. Additionally, the usage of titanium brackets in conjunction with the consumption of fluoridated meals and acidic dentifrice is perfectly safe for such brackets and does not cause corrosion [53].

Discussion

This article includes a summary of orthodontic brackets as well as in-depth descriptions of ceramic, self-ligating, lingual, plastic, butterfly, gold, silver-platinum coated, titanium, and SS brackets. Self-ligating brackets that are already in the sector complement remarkably minimal friction with a secure full bracket engagement to offer a hugely beneficial solution [16]. Additionally, they are robust enough and easy to use to provide the bulk of advantages of this kind of bracket. The cutting-edge elastic slot bracket system's direct torque transfer is made possible by the V slot and V wire [15]. Manufacturers may now construct brackets customized to each patient in order to create a theoretically optimal force system and the required tooth displacement due to new three-dimensional design and manufacturing technology. These brackets will soon become obsolete as technology develops and be replaced by newer ones. When selecting a bracket system, the orthodontist should carefully consider the case at hand and the patient's aesthetic preferences. Table 2 shows a short review of all types of brackets in this article.

**Table 2:** Short review of all types of brackets in this article

Type of bracket	Advantages	Disadvantages
Stainless steel brackets	Affordable, durable, most widely used.	Not aesthetic, demands soldering, high modulus of elasticity, more frequent number of activations, low spring-back than NiTi, decreased corrosion resistance.
Ceramic brackets	Translucent appearance, more strength, increased durability, improved stability in case of color and efficient aesthetic quality.	Lack of ductility, expensive, fragile, stain easily, bulky and a complicated and expensive manufacturing process.
Plastic brackets	Comfortable, cosmetically appealing, less torque deformation.	Not that strong, absence of stiffness, tie wing fracture, irreversible warping, odor, discoloration.
Self-ligating brackets	Various materials available, patients have the option of choosing a metallic as well as ceramic one, regular adhesive or elastic bands are not required, unique shape simultaneously provides the necessary mobility and tension, require fewer force to move teeth, reduced chair-side assistance and appointments, superior overall ergonomics, enhanced control of infection, reduced distress to patients, optimised oral hygiene.	Sophisticated mechanical design, there may be greater occlusal discrepancies and lip pain. More expense, a chance that a clip or slide will break, a larger profile, and difficulties completing as a result of the arch wires' partial expression.
Lingual brackets	Aesthetically appealing, protect teeth from over-calcification.	Speech disruption, occlusal interferences, more bond breakdowns, indirect vision makes it challenging to set brackets accurately, tongue laceration from sharp edges, gingival irritation from plaque build-up and longer chair side time due to the difficulty of inserting and ligating the arch wire.
Butterfly system	More adaptable vertical slots, brackets do not require hooks, convenient and attractive.	Not that accurate hence further enhancements required.
Gold brackets	Eye-catching feature of their appearance, anti-inflammatory and less prone to trigger allergic reactions.	Very expensive, not provided by all orthodontists.
Silver platinum coated brackets	Effective antibacterial activity and tolerance to bio film development.	Expensive, aesthetically not pleasing.
Titanium brackets	Good physical strength and ease of shaping into diverse form. Superior biocompatibility and lower friction.	Plaque build up and discoloration.

## Conclusions

An overview of orthodontic brackets is included in the article, along with in-depth explanations of ceramic, self-ligating, lingual, plastic, butterfly, gold, silver-platinum coated, titanium, and SS brackets. The main research aim of the study was to explore and analyze various types of orthodontic brackets while considering the patient's scenario and aesthetic preferences in selecting the most suitable bracket system. The study provides in-depth explanations of each bracket type, highlighting their unique features, advantages, and disadvantages. It emphasizes the importance of individualizing the bracket choice based on the patient's specific needs. The orthodontist should carefully analyze the scenario at hand and the patient's aesthetic preferences before choosing a bracket system. In conclusion, the research suggests that the bracket system that aligns best with the patient's requirements, whether related to aesthetics, functionality, or other factors, should be the one that is most acceptable and most suitable for them. It is mostly seen that most of the patients prefer either metal or ceramic brackets, out of which the metal brackets are most cost effective and equally efficient in the correction of the architecture of a smile and hence widely used by orthodontists.
